# Sensor-Based Monitoring of Knee Osteoarthritis Symptoms in Free-Living Settings: Scoping Review

**DOI:** 10.2196/84262

**Published:** 2026-07-02

**Authors:** Bo Cui, Bert-Jan van Beijnum, Monique Tabak, Ying Wang

**Affiliations:** 1 Faculty of Electrical Engineering, Mathematics, and Computer Science, Biomedical Signals and Systems Group, University of Twente, De Horst 2, Enschede, Overijssel, 7522 NB, The Netherlands, 31 623160555

**Keywords:** knee osteoarthritis, symptom monitoring, wearable sensors, real-world data, biomechanical assessment, physiological variables, patient-reported outcomes, sensor integration, digital health, personalized medicine

## Abstract

**Background:**

Knee osteoarthritis is a heterogeneous condition characterized by chronic pain, stiffness, and fatigue that fluctuate rapidly over time. Traditional clinical assessments provide only static diagnoses of disease severity, failing to capture the dynamic, day-to-day symptom variability that impacts patient quality of life. While wearable technologies offer the potential for continuous, high-frequency monitoring, previous reviews have examined general technological interventions for knee osteoarthritis management, yet they lack a specific synthesis of technologies for symptom monitoring.

**Objective:**

This study aims to synthesize current research on sensor technologies used for the continuous monitoring of knee osteoarthritis symptoms in free-living or simulated daily environments. Specifically, the review seeks to (1) map sensor modalities to specific symptom domains (biomechanical, physiological, and behavioral); (2) evaluate the alignment between objective sensor metrics and patient-reported outcome measures; and (3) identify gaps in current monitoring paradigms.

**Methods:**

A systematic literature search was conducted across PubMed, Embase, Web of Science, and IEEE Xplore. The review followed the PRISMA-ScR (Preferred Reporting Items for Systematic Reviews and Meta-Analyses extension for Scoping Reviews) guidelines. Eligibility criteria included studies involving participants with knee osteoarthritis using wearable or portable sensors capable of continuous monitoring (eg, inertial measurement units and electrocardiography) and assessing clinical symptoms (eg, pain, fatigue, and stiffness). Studies relying solely on stationary laboratory equipment (eg, force plates) without a portable component were excluded to ensure relevance to real-world applicability. Data regarding sensor types, sampling frequencies, monitored symptoms, and the statistical association between objective features and subjective symptom severity (key findings) were extracted.

**Results:**

A total of 16 studies met the inclusion criteria. The summary constructed from the results revealed a distinct technological saturation: the majority of studies (n=6) used inertial measurement units to quantify biomechanical deficits (eg, gait asymmetry and range of motion), which showed robust correlations with functional limitations. In contrast, there was a notable scarcity of research using physiological sensors (eg, electrocardiography and bioimpedance) to monitor systemic symptoms. Crucially, findings highlighted a significant discrepancy between subjective and objective data, particularly in sleep monitoring, where poor self-reported sleep quality predicted pain exacerbations despite stable objective actigraphy metrics. Furthermore, most systems operated as passive data loggers, with a lack of integration into active feedback loops.

**Conclusions:**

Unlike previous reviews focused solely on biomechanics, this study innovatively maps the use of sensors across a multidimensional symptom spectrum, revealing a critical gap in the monitoring of fatigue and physiological stress. The findings suggest that current sensor applications are limited by a lack of integration with subjective patient experiences. Real-world implementation requires a hybrid monitoring paradigm that combines the ecological validity of wearable sensors with the clinical relevance of patient-reported outcomes. This approach paves the way for digital phenotyping and active feedback systems, offering a personalized strategy for managing the complex symptom burden of knee osteoarthritis.

## Introduction

Knee osteoarthritis is a prevalent degenerative joint disease characterized by the progressive loss of articular cartilage, subchondral bone remodeling, synovial inflammation, and the formation of osteophytes, ultimately leading to joint pain, stiffness, and functional limitations [1]. Knee osteoarthritis primarily affects older adults, with a global prevalence estimated at 10%‐15% among individuals aged 60 years and older [2]. Beyond structural joint damage, the disease is clinically defined by its debilitating symptoms, primarily pain, stiffness, tenderness, and swelling [3]. These symptoms are not static; rather, they fluctuate daily or even hourly, influenced by physical activity, weather, and psychological factors [4,5]. This variability significantly impacts patients’ mobility and quality of life, necessitating timely and dynamic management strategies [6].

Traditional assessment methods, such as clinical physical examinations and self-reported questionnaires (patient-reported outcome measures [PROMs]), remain the gold standard for evaluating these symptoms [3,4]. However, these subjective measures are limited by recall bias and typically provide only a snapshot of the patient’s condition during clinical visits, often failing to capture the continuous, real-world variability of knee osteoarthritis symptoms [7,8]. To overcome these limitations, objective and longitudinal data are needed to reflect the patient’s actual daily status. Wearable sensing technologies offer a robust solution by enabling the continuous collection of physiological and biomechanical data outside of clinical settings [9,10].

Importantly, these technologies enable the collection of objective digital markers that are increasingly explored for monitoring and characterizing osteoarthritis symptoms in daily life [11,12], complementing rather than replacing patients’ subjective reports. Although sensors do not measure pain or stiffness directly, they provide quantifiable metrics that track the physiological and biomechanical consequences of these symptoms. For instance, published evidence suggests that heightened knee pain often manifests objectively as reduced gait speed, altered gait symmetry [11], or elevated physiological stress markers [13,14], whereas joint stiffness is typically associated with restricted range of motion (ROM) or specific kinematic deviations during daily activities [12]. Identifying and validating these specific digital markers is the fundamental step for developing future monitoring systems that can infer symptom status without burdening patients with constant manual reporting.

However, the current landscape of these digital markers remains fragmented. While various sensor-derived metrics exist, it is often unclear which specific combinations of markers effectively capture the multidimensional nature of knee osteoarthritis symptoms in a free-living environment. Most existing monitoring approaches leverage only a subset of available technologies, lacking a comprehensive evidence base regarding their association with specific symptoms [10,15,16]. Therefore, a comprehensive synthesis of the current state-of-the-art digital markers is needed to support the development of technologies capable of more accurately capturing daily symptom fluctuations and reflecting the patient’s multidimensional symptom profile.

Previous reviews have examined general technological interventions for knee osteoarthritis management [15,16], yet they lack a specific synthesis of biomarkers for symptom monitoring. Therefore, this scoping review aims to map the existing literature to identify objective digital markers that represent knee osteoarthritis symptoms in free-living settings. Specifically, we explore the evidence linking these objective sensor-derived metrics to subjective knee osteoarthritis symptoms across a spectrum of settings, ranging from laboratory-based validation studies to continuous monitoring in free-living environments. Therefore, our specific research objectives are to (1) map available sensor modalities to a multidimensional spectrum of clinical symptoms, including underresearched domains like fatigue, sleep quality, and physiological stress; (2) evaluate the current evidence regarding the alignment or discrepancy between objective digital markers and subjective PROMs; and (3) identify which digital markers are the most promising candidates to guide the development of future hybrid monitoring paradigms.

## Methods

### Overview

The scoping review was conducted following the PRISMA-ScR (Preferred Reporting Items for Systematic Reviews and Meta-Analyses extension for Scoping Reviews) guidelines [17].

### Search Strategy

To ensure a comprehensive identification of relevant literature, we developed a systematic search strategy in accordance with the PRISMA-S (Preferred Reporting Items for Systematic Reviews and Meta-Analyses and search extension) guidelines [18]. We expanded our search beyond standard biomedical databases to include engineering and technology-focused repositories to capture sensor-based research. The electronic databases searched included PubMed, Embase, Web of Science, and IEEE Xplore.

The search strategy was designed to capture the intersection of 4 key concepts. First, we defined the population, specifically targeting patients with knee osteoarthritis. Second, we identified the target symptoms, which included pain, stiffness, fatigue, and ROM. Third, we established the monitoring context, focusing on daily, continuous, or ambulatory settings. Finally, we specified the technology, encompassing wearable sensors, mobile apps, and physiological signals.

The search string used a combination of medical subject headings and free-text keywords, including specific sensor modalities (eg, “accelerometry,” “photoplethysmography,” and “electromyography”) and digital tools (eg, “smartphone applications”), as detailed in the *Introduction* section. The search was updated and re-run on February 5, 2026. To ensure the review focused on contemporary sensor technologies relevant to current clinical practice, the search was limited to studies published in the last 5 years (from January 1, 2019, to the search date). This timeframe was selected to align with the rapid proliferation of wearable technology and smartphone integration in health care. The full search terms are reported in Multimedia Appendix 1 and were adapted for the specific requirements of each database.

To fully adhere to the PRISMA-S reporting guidelines, we explicitly state the following methodological details regarding our search process: the search strategy was developed collaboratively by the research team but was not formally peer-reviewed by an independent medical librarian (PRESS [Peer Review of Electronic Search Strategies]). No specific methodological search filters (eg, study design filters) were applied, nor were the search strategies adapted from prior literature reviews. Additionally, our search was restricted to the above databases; we did not search clinical trial registries, browse unindexed online resources, or contact authors and manufacturers for additional data, as our focus was strictly on peer-reviewed published evidence. To address potential gaps in electronic indexing and maximize coverage, we also performed a manual hand-search of the reference lists of included studies and relevant systematic reviews to identify additional eligible articles. Furthermore, regarding other search specifics, all databases were searched independently on their respective platforms; no simultaneous multidatabase searching via a single platform was performed. No additional information sources or search methods beyond those explicitly described were used. Additionally, no formal update search protocols or automated email alerts were implemented throughout the review process.

All identified citations were collated and uploaded into EndNote (Clarivate Analytics), and duplicates were removed. Specifically, the deduplication process involved using EndNote’s automated “Find Duplicates” function, followed by a manual verification by the reviewers to ensure accuracy. To minimize selection bias and ensure rigor, the screening process was conducted in two distinct stages. In the first stage, which involved title and abstract screening, 2 independent reviewers (BC and YW) evaluated the records against the predefined inclusion criteria. In the second stage, representing the full-text review, potentially eligible articles were retrieved and assessed in detail for final inclusion. Any disagreements between the reviewers at either stage were resolved through discussion or, if necessary, consultation with a third reviewer to reach a consensus.

### Eligibility Criteria

Studies were selected based on the following inclusion and exclusion criteria, explicitly designed to identify technologies suitable for the objective, ecological monitoring of symptom-related variables. For the population, studies needed to focus on adults diagnosed with knee osteoarthritis. Regarding technology and feasibility, the study had to use noninvasive wearable sensors (eg, IMUs and smartwatches) or mobile health technologies (eg, smartphone apps) that are portable and capable of unsupervised data acquisition without requiring constant health care professional involvement, thereby ensuring applicability for free-living monitoring. Conceptually, the study needed to investigate objective biomechanical or physiological digital markers and explicitly assess their association with subjective symptoms. In terms of context, we included studies conducted in free-living environments (home or community) or laboratory settings, provided that the laboratory studies simulated daily living activities (eg, level walking and sit-to-stand tasks) to validate the sensor’s potential for real-world application. Finally, eligible study designs were restricted to original quantitative or qualitative research published in English.

Conversely, studies were excluded based on several criteria. Regarding the population, studies were excluded if participants had undergone total knee arthroplasty, ensuring our focus remained on monitoring natural disease progression. We also excluded studies based on technological constraints; specifically, if the monitoring method relied on stationary clinical infrastructure (eg, optoelectronic motion capture systems and nonportable force plates) that precludes ecological integration into free-living environments, or if it relied solely on manual clinical assessment tools (eg, hand-held dynamometers, visual inspection scales, or manual goniometry performed by a therapist), as these do not represent automated, continuous monitoring technologies. Furthermore, regarding intervention focus, studies were excluded if their primary aim was to evaluate therapeutic interventions, such as rehabilitation programs, pharmacological clinical trials, acupuncture, or radiological diagnostics, unless sensor-derived symptom markers were explicitly investigated as a primary outcome. Finally, publication types such as literature reviews, study protocols, editorials, or conference abstracts without available full texts were excluded.

### Data Charting Process

Data extraction was conducted using a standardized data charting form developed specifically for this review. To ensure consistency and rigor, the form was pilot-tested on a random sample of 5 studies. Data were extracted by one reviewer (BC) and verified by a second reviewer (YW) for accuracy. Any discrepancies were resolved through discussion.

### Data Items

We extracted data at two levels: study characteristics and specific monitoring variables. Specifically, the extracted study characteristics included the author, year, country, study design, setting (laboratory vs free-living), and participant demographics such as sample size, age, sex, and BMI. Regarding the specific monitoring variables, we systematically categorized the data into 3 main areas. First, for monitoring technology, we recorded the sensor type (eg, IMU, electromyography [EMG], and photoplethysmography [PPG]), body placement (eg, lumbar, shank, and wrist), sampling frequency, and duration of monitoring. Second, for symptom assessment, we extracted the method of symptom quantification (eg, VAS, WOMAC, and NRS), the specific symptom domains measured (such as pain, stiffness, and fatigue), and the frequency of data collection (eg, momentary, daily, or weekly). Finally, we documented the key findings, focusing on the reported statistical associations between the objective sensor-derived variables and the subjective symptom scores.

### Critical Appraisal of Evidence

As this is a scoping review aiming to map available literature rather than assess efficacy, a formal risk of bias assessment (eg, Cochrane ROB) was not performed. However, to address the rigor of the included evidence, we classified studies based on their design (eg, proof-of-concept, validation study, and longitudinal monitoring) and setting (controlled laboratory vs unsupervised free-living environment). This classification allows for a nuanced understanding of the current maturity of sensor-based symptom monitoring.

### Data Synthesis and Categorization

In accordance with JBI (Joanna Briggs Institute) guidelines for scoping reviews, the extracted data were analyzed descriptively. We used simple frequency counts to summarize study characteristics, sensor modalities, and targeted symptom domains. Furthermore, a basic qualitative content analysis was applied to map and categorize the findings into distinct physiological and biomechanical domains, identifying overarching trends and evidence gaps. We synthesized the results narratively, grouping studies based on the primary physiological or biomechanical domain targeted by the sensor. To align with the extracted data, the findings were categorized into five functional domains. First, we identified biomechanical and functional changes, which encompass gait kinematics (eg, speed and stride length), joint angles, and functional movement patterns measured by motion sensors. Second, the domain of neuromuscular control and postural stability includes muscle activity patterns (eg, surface electromyography [sEMG]), postural sway metrics, and balance parameters that serve as proxies for pain-induced inhibition or instability. Third, we grouped physiological markers, capturing autonomic responses such as heart rate variability (HRV), skin temperature, and electrodermal activity, used to infer pain-related stress. Fourth, physical activity patterns were categorized to include gross activity levels, step counts, and energy expenditure reflecting functional capacity. Finally, the fifth domain addresses sleep and circadian interactions, detailing actigraphy-derived sleep quality metrics and their temporal relationship with symptom fluctuations.

Furthermore, to visualize the extent and nature of the available literature, we constructed an Evidence Gap Map. This synthesis matrix cross-referenced the identified sensor modalities against the five clinical symptom domains, enabling the systematic identification of areas with technological saturation versus those with critical evidence gaps.

## Results

### Literature Search and Selection

**Figure 1.**
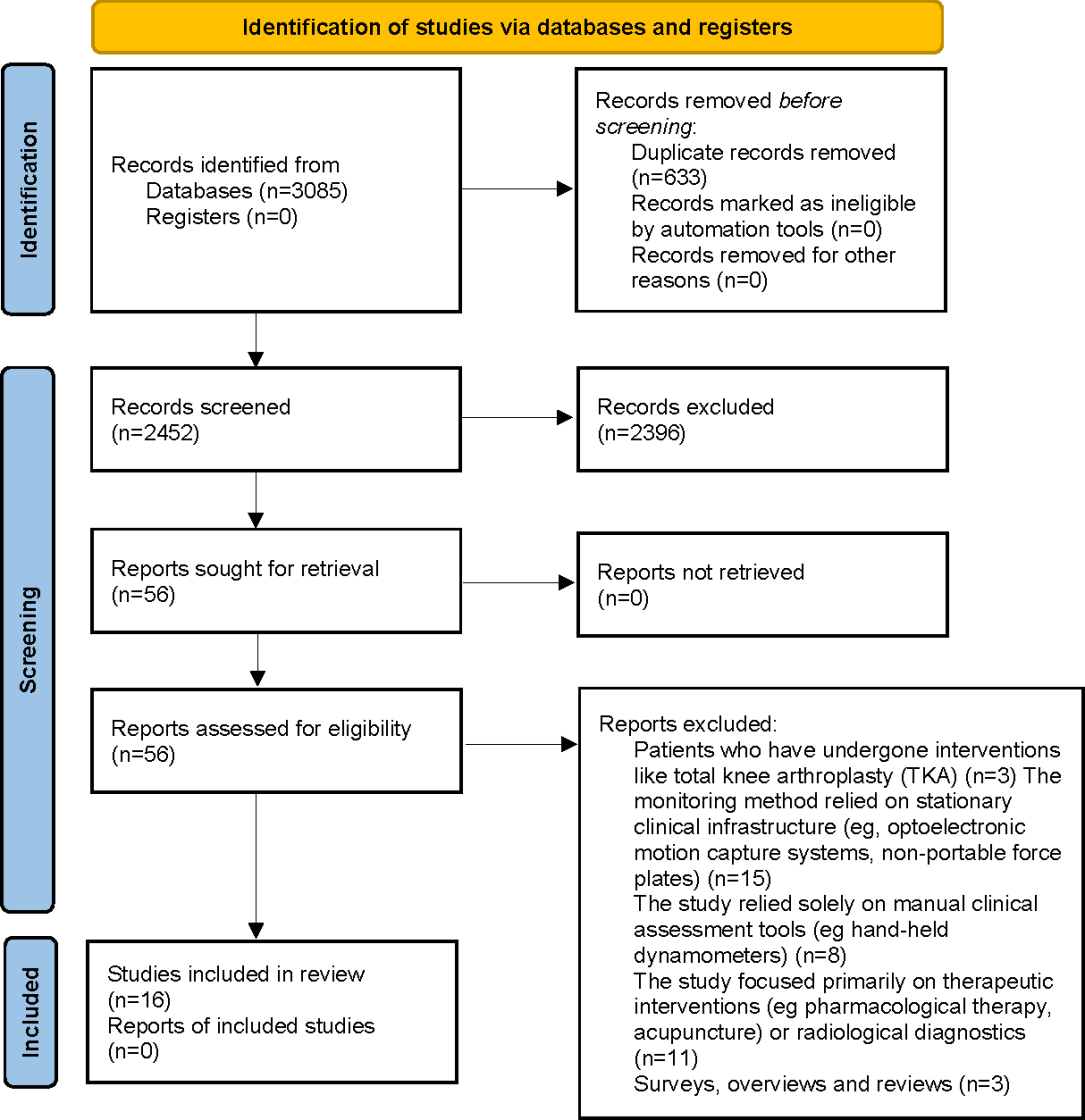
PRISMA (Preferred Reporting Items for Systematic Reviews and Meta-Analyses) flow diagram.

The search strategy and selection process were conducted in accordance with the PRISMA-S [18]. Figure 1 illustrates the PRISMA flow diagram. The comprehensive electronic search across multiple databases yielded a total of 3085 records. Following the removal of duplicates, 2452 unique records remained for title and abstract screening. Of these, 2396 records were excluded as they did not meet the primary inclusion criteria based on the title and abstract review.

Consequently, 56 full-text articles were retrieved and assessed for eligibility. At this stage, 40 studies were excluded. To strictly follow the feasibility requirements for free living monitoring, studies relying solely on nonportable, stationary laboratory equipment (eg, force plates and isokinetic dynamometers) or those lacking direct correlation between sensor data and clinical symptoms were excluded. Finally, a total of 16 studies [19-34] met all inclusion criteria and were included in this scoping review.

### Study Characteristics and Rigor of Evidence

#### Study Design and Setting

Table 1 summarizes the characteristics of the included studies. Regarding the rigor and setting of the evidence, the studies were categorized into two distinct environments. A total of 11 studies [19-23,25,28,30,31,33,34] were conducted in controlled laboratory settings, using cross-sectional designs to validate sensor metrics against gold standards or to differentiate between symptoms during simulated daily tasks (eg, walking and stair climbing). In contrast, 5 studies [24,26,27,29,32] used free-living protocols, monitoring participants in their home environments for durations ranging from 5 days to 12 weeks. These longitudinal studies primarily focused on capturing symptom fluctuations and functional decline over time, offering higher ecological validity but often lacking simultaneous ground-truth validation.

**Table 1.** Summary of characteristics for the 16 included studies evaluating sensor technologies for knee osteoarthritis. Data detail the specific sensor modalities used (eg, IMU^a^, EMG^b^, ECG^c^), sensor placement, duration of monitoring in free-living settings, and the alignment between objective sensor metrics and subjective clinical symptoms (eg, pain, fatigue, and stiffness) (Multimedia Appendix 2).

Category	Study information and design				Population				Technology and protocol				Symptom assessment			Objective markers and key findings	
	Year	Country	Study design	Environment	Sample size, n	Age (y), mean (SD)	BMI (kg/m^2^), mean (SD)	Sex (female), n	Sensor	Placement	Sampling rate (Hz)	Duration	Tool	Symptom	Frequency	Marker	Main finding
Biomechanical and Functional Changes [19]	2022	Japan	Cross-sectional Study	Lab	20	60 (5.79)	23.9 (3.26)	15	RGB-D^d^	Knee joint	20	One-time capture	VAS^e^	Pain	One-time	Knee joint trajectory	Early patients with KOA^f^ showed RKJT^g^ was 10 mm larger than the control group (*P*=.04), correlating with pain severity.
Biomechanical and Functional Changes [20]	2021	Japan	Cross-sectional Study	Lab	21	24.9 (3.4)	72.1 (7.10)	17	IMU	Full-body	60	5 s	VAS	Pain	One-time	Knee flexion excursion, muscle strength	As walking speed increased, stride length and knee flexion excursions significantly increased. Knee flexion score correlated significantly with pain score and muscle strength.
Biomechanical and Functional Changes [21]	2022	USA	Cross-sectional Study	Lab	25	64 (07)	29 (04)	13	IMU	Lumbar	128	2 min	KOOS^h^	Pain	One-time	Gait parameters	Gait velocity, cadence, step count, and stride length were the most important features for classifying KOA versus pain levels.
Biomechanical and Functional Changes [22]	2024	USA	Cross-sectional Study	Lab	28	68.7 (2.4)	31.5 (1.6)	18	Cleveland Clinic configuration marker	Full-body	200	One-time capture	WOMAC^i^	Pain	One-time	vGRF^j^	Higher peak tibial acceleration is linked to worse knee pain (*r*=0.39; *P*=.01) and higher vertical load rates.
Biomechanical and Functional Changes [23]	2024	USA	Cross-sectional Study	Lab	42	60.4 (12.6)	30 (6.8)	28	IMU	Tibia	1000	3 min	VAS	Pain	One-time	PTA^k^	An association exists between pain, obesity, and increased ankle-joint loading during the weight-acceptance phase of stair descent (*P*<.001).
Biomechanical and Functional Changes [30]	2020	Belgium	Cross-sectional Study	Lab	19	65.1 (5.2)	26 (2.2)	7	IMU	Lower limb joints	60	One-time capture	KOOS	Pain, ADL^l^	One-time	Joint angles	IMU system successfully discriminated patients with KOA from controls in all tasks except sit-to-stand. Patients with KOA showed reduced knee flexion ROM^m^ across walking, lunges, squats, and stairs (*P*=.001).
Neuromuscular Control and Postural Stability [24]	2025	Greece	Cohort Study	Daily life	21	51 (8)	—^n^	13	sEMG	Quadriceps	2000	8 weeks	WOMAC	Pain	Daily	Muscle performance	Significant Group × Time interaction for RMS (*P*<.001). Increase in sEMG^o^ RMS^p^ (activation) coincided with a significant decrease in WOMAC pain scores.
Neuromuscular Control and Postural Stability [25]	2021	USA	Cohort Study	Lab	1666	67.2 (7.6)	32 (13)	985	sEMG	Quadriceps and hamstrings	1000	One-time capture	WOMAC	Pain	One-time	Muscle Coactivation	Significant inverse associations between hamstring coactivation and quadriceps strength. Lower quadriceps strength predicts incident KOA and pain.
Neuromuscular Control and Postural Stability [34]	2025	Malaysia	Cross-sectional Study	Lab	64	69 (4)	25.92 (3.25)	19	sEMG	Lower limb joints	9M	One-time capture	Ultrasonic system	Stiffness	One-time	Muscle Coactivation Index	Quadriceps stiffness was significantly greater in the KOA group and correlated with functional deficits.
Physical Activity Patterns [26]	2022	China	Cohort Study	Daily life	65	61.3 (5.99)	28.7 (28.66)	30	IMU	Wrist	60	7 days	WOMAC	Pain	Daily	Steps	Weak but significant correlation between change in mean steps per day and global improvement/WOMAC function (*P*=.08).
Physical Activity Patterns [27]	2024	UK	Proof of Concept	Daily life	38	58 (9)	—	33	IMU	Wrist	—	12 weeks	MSK-HQ^q^	Pain	Daily	Activity level	Significant improvements across all symptom domains (*P*<.001). Largest effect sizes observed for fatigue (*d*=1.30) and day pain (*d*=1.03) following intervention.
Physiological Markers [28]	2024	Germany	Cross-sectional Study	Lab	148	66 (27)	30.6 (5.8)	68	ECG	Chest	60	5 min	PSQ-20^r^	Pain	One-time	Heart rate variability	Weak negative correlation between HRV^s^ and pain. WOMAC pain significantly correlated with Cortisol (positive) and DHEA-S (negative).
Physiological Markers [29]	2023	USA	Cohort Study	Daily life	10	73.5 (8.26)	—	9	Bioimpedance sensing	Knee joint	64	7 days	ESM	Pain	Daily	Bioimpedance	Results suggest bioimpedance metrics can be used as a predictor for active pain experiences in knee osteoarthritis.
Physiological Markers [31]	2022	USA	Cross-sectional Study	Lab	30	58.3 (9.3)	33.5 (5.8)	20	Portable gas exchange system	Face	10	10 min	KOOS	Pain, fatigue	One-time	VO_2 peak, heart rate, energy cost	Higher energy cost for walking is linked to reduced physical activity. Higher fatigue and fatigability mediated the associations between walking energetics and activity.
Physiological Markers [33]	2020	Brazil	Cross-sectional Study	Lab	11	63.1 (9.5)	28.7 (4)	—	Infrared sensor	Thigh, leg	30	One-time capture	WOMAC, VAS	Physical function, pain	One-time	Temperature	Affected knees had higher temperature, though not directly associated with pressure pain thresholds in this small sample.
Sleep and Circadian Interactions [32]	2019	USA	Cohort Study	Daily life	160	71 (4)	29.6 (3.4)	99	IMU	Wrist	60	5 days	WOMAC, BFI	Pain, fatigue, sleep	Daily	Activity level, sleep duration	Contrast between subjective and objective data. Better subjective sleep predicted lower pain (*P*<.001), but objective metrics showed no meaningful association with daily pain fluctuations.

a IMU: inertial measurement unit.

b ESM: experience sampling method.

c ECG: electrocardiography.

d RGB-D: red green blue-depth.

e VAS: Visual Analog Scale.

f KOA: knee osteoarthritis.

g RKJT: range of the knee joint trajectory.

h KOOS: knee injury and osteoarthritis outcome score.

i WOMAC: Western Ontario and McMaster Universities Osteoarthritis Index.

j vGRF: vertical ground reaction force.

k PTA: peak tibial acceleration.

l ADL: activities of daily living.

m ROM: range of motion.

n Not available.

o sEMG: surface electromyography.

p RMS: root mean square.

q MSK-HQ: Musculoskeletal Health Questionnaire.

r PSQ-20: Perceived Stress Questionnaire.

s HRV: heart rate variability.

#### Participant Demographics

Sample sizes varied significantly depending on the study design, ranging from 10 to 1666 participants. Studies using complex multisensor setups or detailed biomechanical profiling generally involved smaller sample sizes (n<30) [19-22,24,25,31,33], whereas longitudinal investigations using commercial wearable sensors or devices (eg, accelerometers and smartwatches) incorporated larger cohorts [23,26-30]. The demographic distribution reflected the typical knee osteoarthritis population, with a notable prevalence of female participants (approx. 58.2%) and individuals classified as overweight or obese (BMI>25 kg/m²), which was consistently reported as a factor influencing both symptom severity and sensor-derived gait parameters. Geographically, the majority of studies were conducted in the United States (n=7) [21-23,26,31,32,34], followed by Japan (n=2) [19,20].

#### Distribution of Sensor Modalities and Symptoms

**Figure 2.**
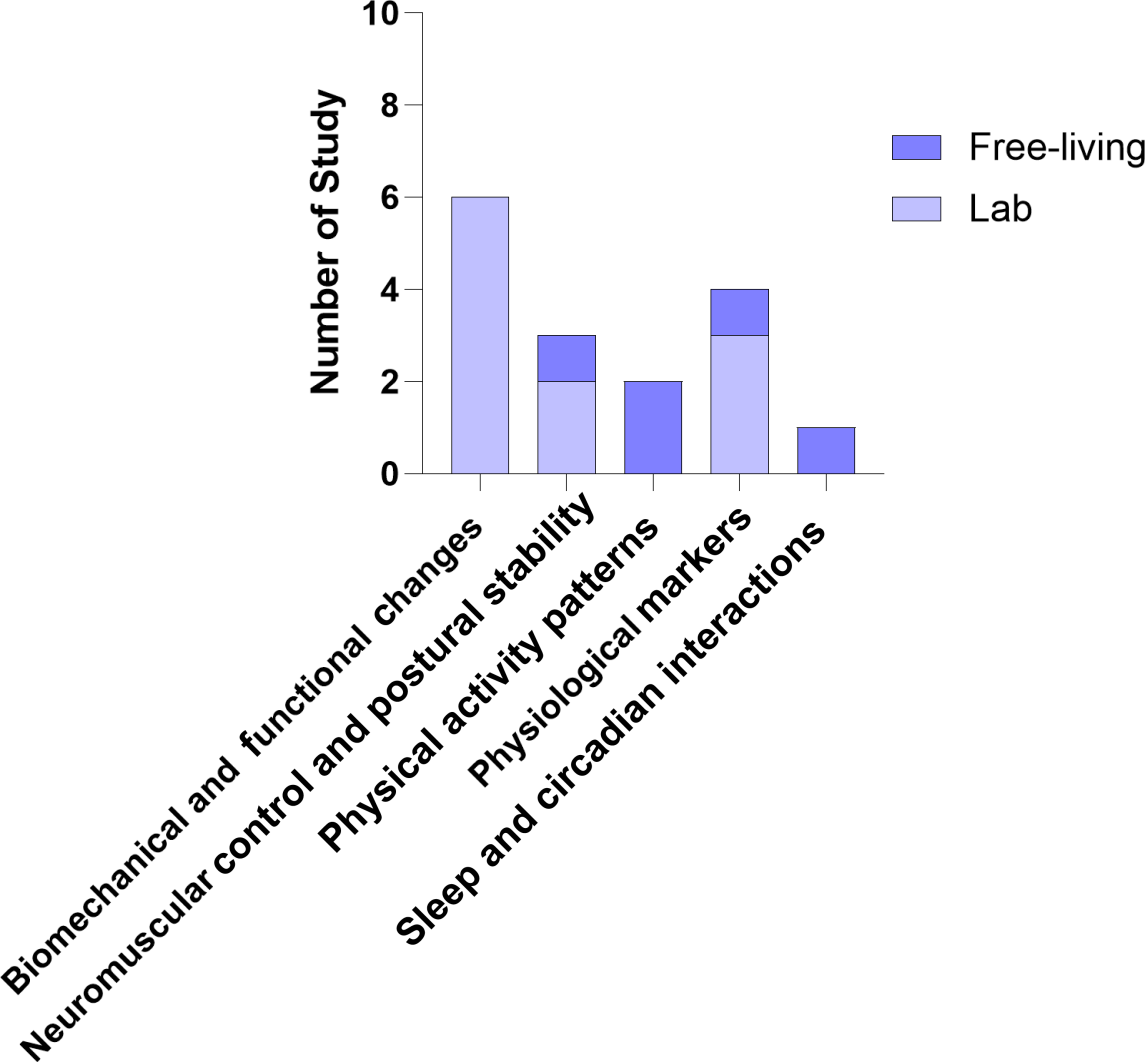
Distribution of sensor modalities across study environments. A stacked bar chart illustrating the total number of included studies categorized by primary sensor domains. The color segments represent the study setting, highlighting the use of laboratory environments (light purple) compared to free-living environments (light blue). The chart visualizes the environmental gap, particularly the reliance on laboratory settings for biomechanical assessments.

Overall, the descriptive analysis revealed a strong concentration of research in specific domains, alongside notable evidence gaps. Out of the 16 included studies [19-34], the majority used biomechanical monitoring (n=6) [19-23,30] via inertial measurement units (IMUs), focusing on kinematics, targeting functional limitations and gait abnormalities. In contrast, physiological monitoring (eg, measuring inflammation via temperature or stress via HRV) (n=4) [28,29,31,33] and neuromuscular monitoring (n=3) [24,25,34] were markedly underrepresented, representing emerging but less explored domains in recent years. Furthermore, our frequency counts highlighted a methodological trend: while all studies aimed to explore free-living symptom monitoring, a significant proportion (11 out of 16) still relied on lab-simulated tasks rather than continuous multiday monitoring. As illustrated in Figure 2, this environmental disparity is particularly evident within biomechanical and neuromuscular research, where laboratory settings overwhelmingly dominate. In contrast, sleep and physical activity monitoring are exclusively conducted in free-living environments. Regarding symptom assessment, pain was the most frequently evaluated clinical outcome across all categories (n=14). Interestingly, pain was correlated with diverse digital markers ranging from joint angles to sleep efficiency, highlighting the multidimensional potential of sensor-based assessment.

#### Evidence Mapping and Gaps

**Figure 3.**
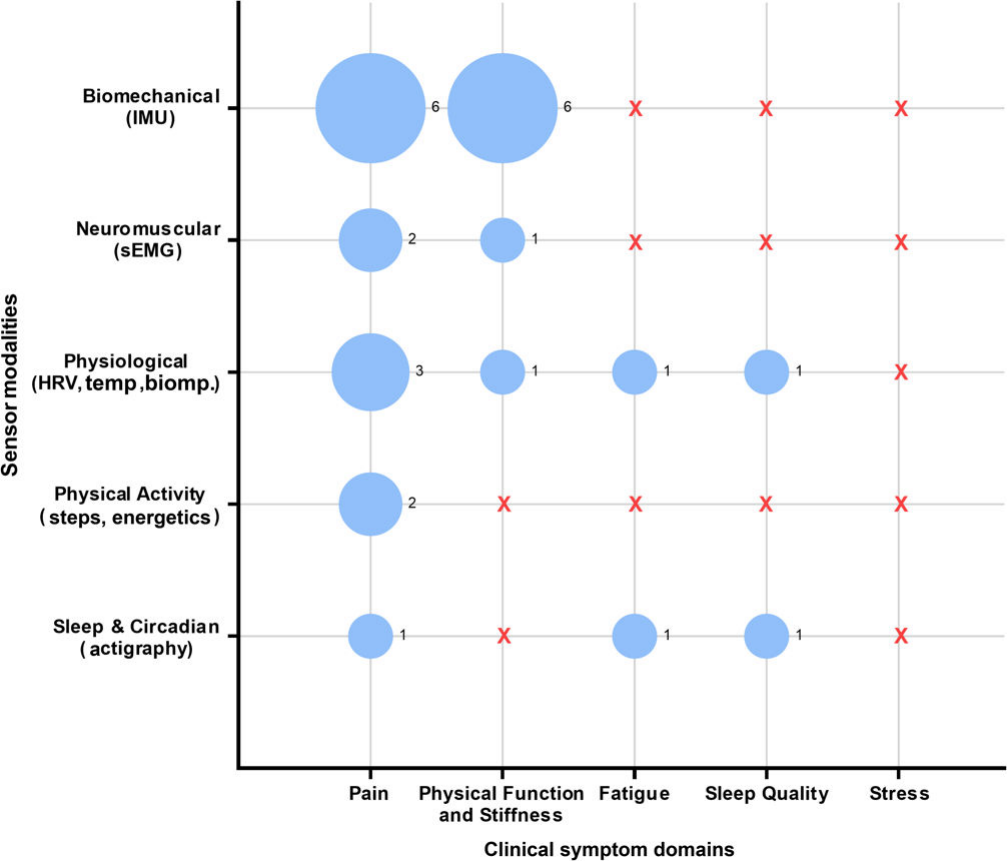
Evidence gap map illustrating the distribution of research on sensor-based monitoring for knee osteoarthritis (n=16 unique studies). The matrix cross-references sensor modalities (y-axis) with clinical symptom domains (x-axis). Bubble size is proportional to the number of included studies, highlighting a saturation of research in biomechanical gait analysis (inertial measurement units). Red “X” markers denote “evidence gaps,” indicating a scarcity of studies using physiological sensors or monitoring sleep and stress in free-living settings. The total sum of data points across all bubbles exceeds 16 because several studies used multiple sensor modalities or assessed multiple clinical symptom domains simultaneously. sEMG: surface electromyography; HRV: heart rate variability; IMU: inertial measurement unit; temp: temperature; biomp: bioimpedance.

To visualize the distribution of research efforts, an evidence gap map (Figure 3) was constructed. The map reveals a clear concentration of evidence linking biomechanical sensors (IMUs) to pain and functional limitations, representing the most mature area of research. In contrast, significant gaps remain in the application of physiological sensors (eg, HRV and bioimpedance) and activity level to monitor subjective symptoms such as fatigue, sleep quality, and stress. While pain is the most frequently assessed symptom across all sensor modalities, the multidimensional nature of osteoarthritis symptoms—particularly the interplay between physiological stress and fatigue—remains under-investigated in the current sensor-based literature.

**Figure 4.**
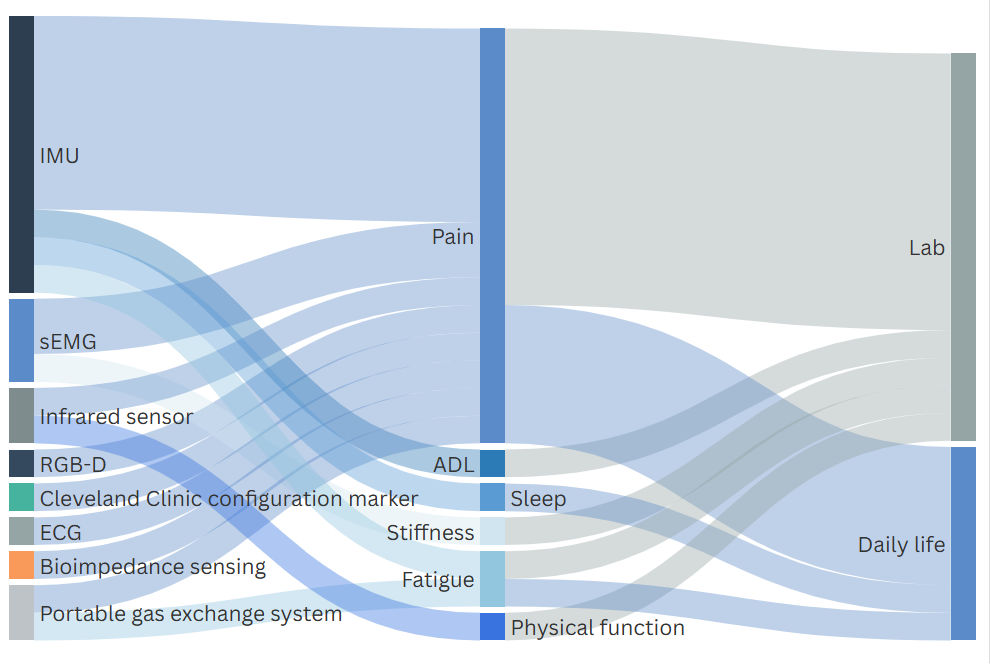
Sankey diagram mapping sensor modalities to monitored symptoms and study environments. The alluvial flow pathways illustrate the connections between the types of wearable sensors used (left), the specific clinical symptoms assessed (center), and the environment in which the monitoring occurred (right). The width of the bands is proportional to the number of studies. ADL: activities of daily living; ECG: electrocardiogram; sEMG: surface electromyography; IMU: inertial measurement unit; RGB-D: red green blue plus depth.

To further explain the complex, multilayered relationships among the used technologies, targeted symptoms, and specific study settings, a Sankey diagram (Figure 4) was constructed. The flow pathways visually confirm that while ’pain’ is the central node connecting nearly all sensor types, the downstream application reveals a critical bottleneck: the thickest pathways originating from IMUs and sEMG frequently terminate in “Lab” settings. Conversely, pathways reaching the “Daily life” environment are sparser and supported by actigraphy or simplified commercial wearables.

#### Biomechanical and Functional Changes

##### Overview

Wearable IMUs and camera-based systems were the predominant technologies for quantifying movement quality. Studies consistently identified that individuals with knee osteoarthritis exhibit distinct kinematic alterations compared to healthy controls, specifically reduced knee flexion ROM during the swing phase of walking and stair climbing [19,20,30]. Crucially, this sensor-measured reduction in flexion was not merely a mechanical deficit but was significantly associated with higher self-reported pain intensity [19,20], suggesting that stiffened gait patterns serve as a protective mechanism against pain.

Beyond joint angles, impact loading was a critical metric. Accelerometers mounted on the tibia revealed that higher peak tibial acceleration during walking was positively correlated with both instantaneous and vertical load rates, which in turn were linked to worse knee pain severity [22,23]. Furthermore, sensors placed on the lumbar region detected compensatory movements, such as reduced trunk and pelvic rotation, which correlated with functional decline in daily tasks [21].

In summary, while biomechanical monitoring represents a mature technological domain, current evidence indicates that it predominantly captures the physical consequences and compensatory mechanics of knee osteoarthritis, highlighting a need to integrate these metrics with simultaneous subjective symptom reporting (a hybrid approach) for comprehensive monitoring.

##### Neuromuscular Control and Postural Stability

Assessment of neuromuscular function shifted from static strength testing to dynamic monitoring of muscle activation and balance. sEMG studies highlighted that altered muscle coactivation patterns—specifically the simultaneous contraction of quadriceps and hamstrings (measured by the TMCf index)—were synchronous with increased pain scores during functional tasks [25,34]. Longitudinal monitoring further demonstrated that rehabilitation-induced increases in sEMG root mean square values (indicating improved muscle activation) paralleled significant reductions in WOMAC pain and stiffness scores [24].

Regarding postural stability, smartphone-based accelerometers worn on the lower back effectively quantified postural sway. Increased sway magnitude (root mean square) during static standing was identified as a sensitive marker for impaired neuromuscular control, showing a significant positive correlation with TUG test duration and fall risk in end-stage patients with knee osteoarthritis [24].

Collectively, these findings suggest that neuromuscular sensors are highly valuable for detecting subtle pain-induced functional inhibition; however, a significant gap remains, as their application is still largely confined to short-duration, structured tasks rather than continuous free-living monitoring.

##### Physiological Markers

Sensors targeting physiological signals provided objective insights into the internal inflammatory and autonomic state of the patient with knee osteoarthritis. HRV, derived from wearable electrocardiography (ECG) sensors, was found to be significantly lower in patients with late-stage osteoarthritis compared to early-stage osteoarthritis. This reduction in HRV correlated with higher chronic stress markers (eg, cortisol/DHEA-S ratio) and elevated WOMAC pain scores, linking autonomic dysregulation to the experience of chronic pain [28].

Locally, knee bioimpedance sensors demonstrated the capacity to discriminate between edematous and healthy tissue. Changes in tissue impedance were predictive of active pain experiences and self-reported swelling [29]. While infrared thermal sensors detected elevated skin temperature in affected knees, one study noted that this thermal increase did not directly correlate with pressure pain thresholds in a small cohort, suggesting that temperature may reflect inflammation rather than immediate pain sensitivity [33].

Overall, while physiological markers offer a promising window into the systemic and local inflammatory stress associated with knee osteoarthritis, the notable scarcity of studies in this area highlights a critical evidence gap in multimodal sensor integration for holistic symptom assessment.

##### Physical Activity Patterns

The relationship between sensor-measured physical activity and symptoms appeared complex. Portable metabolic systems revealed that individuals with knee osteoarthritis exhibited a higher energy cost of walking (VO_2), and this reduced energetic efficiency was significantly associated with higher reported fatigue levels [31].

Regarding daily activity volume, results from wrist-worn accelerometers were mixed. While some longitudinal data suggested that increased daily step counts were weakly associated with improvements in WOMAC function over time [26], other studies indicated that higher intensity activity could trigger immediate pain exacerbations in specific subgroups [27]. This discrepancy highlights that the quality of movement (eg, energetics, impact load) may be more closely linked to symptom burden than the total quantity (step count) of movement.

This specific discrepancy highlights a crucial overarching trend: measuring the biomechanical quality and impact load of physical activity may be far more clinically relevant for symptom monitoring than merely tracking gross activity volume (eg, total step counts).

##### Sleep and Circadian Interactions

Longitudinal monitoring using wrist actigraphy revealed a critical discrepancy between perceived and physiological sleep metrics. Subjective sleep quality was identified as a robust predictor of next-day symptoms, with poorer self-reported sleep preceding days of higher morning pain and fatigue (*P*<0.001) [32]. However, objective sensor metrics (eg, total sleep duration, wake after sleep onset) often failed to show clinically meaningful associations with daily fluctuations in pain or fatigue, despite statistical significance in some models [32]. This suggests that current actigraphy algorithms may not fully capture the qualitative aspects of sleep disturbance (eg, fragmentation, micro-arousals) that are most relevant to the pain experience in knee osteoarthritis.

## Discussion

### Principal Findings

This scoping review synthesized the current evidence on sensor technologies for monitoring knee osteoarthritis symptoms in free-living settings. Relative to our initial objective of evaluating the feasibility and validity of these technologies, our primary finding—visualized in the evidence gap map (Figure 3)—is a distinct technological imbalance. Research is heavily saturated with biomechanical studies using IMUs to quantify gait and movement deficits [20,21,23,26,27,30,32]. In contrast, there is a notable lack of investigations using physiological sensors (eg, ECG and bioimpedance) to monitor systemic symptoms such as fatigue, stress, and sleep quality [28,29,31,32]. While biomechanical markers have demonstrated robust discrimination between patients with osteoarthritis and healthy controls [19], the link between physiological sensor data and patient-reported symptom fluctuations remains an emerging field requiring further validation.

### Interpretations and Clinical Implications

While the primary focus of this review is sensor-based monitoring, discussing PROMs is methodologically and clinically unavoidable. At present, no noninvasive sensor can directly quantify a subjective experience (such as pain or fatigue) without patient input. Therefore, PROMs remain the indispensable ground truth against which all objective sensor metrics must be validated [3,4]. However, our review reveals two critical limitations of relying solely on subjective reports. First, subjective perception may diverge from physiological reality. A prime example from our review is the observation in sleep monitoring: while subjective sleep quality strongly predicted morning pain, objective actigraphy metrics (eg, sleep duration) did not [32]. This discrepancy is clinically revealing: it suggests that for some patients, the perception of poor sleep (distress) drives pain more than the actual physiological lack of sleep. Without objective sensor data to contrast with the subjective report, a clinician might incorrectly prescribe sedatives for insomnia, whereas the sensor data would reveal that the patient is sleeping but unrefreshed.

Furthermore, historically, pain was the primary outcome for knee osteoarthritis symptoms management [1], which can also be seen from Table 1, with the vast majority of included studies focusing on pain as the major symptom outcome. However, our review highlights that fatigue, stiffness, and sleep disturbances are equally debilitating contributors to disability [4]. Subjective recall of these fluctuating symptoms may be biased and varies between patients [8]. Sensors offer a unique advantage here: they can continuously quantify symptom drivers, such as micro-arousals in sleep (Actigraphy) [32] or elevated muscle coactivation (sEMG) [24,25,34], before they manifest as conscious pain. This evidence from our review demonstrates that objective sensor metrics and subjective reports provide different and additive information. Therefore, rather than viewing sensors as a complete replacement for PROMs, we advocate for a necessary hybrid approach. In this paradigm, continuous passive monitoring via sensors can successfully bridge the temporal gaps between clinical visits, significantly reducing the patient’s active reporting burden (eg, the need for highly frequent questionnaire inputs), while periodic PROMs continue to capture the essential subjective ground truth. Indeed, several studies included in this review have already laid the groundwork for this by successfully pairing sensor data with standard subjective questionnaires (eg, WOMAC, VAS, and KOOS) [19,25,30]. Building upon this existing foundation, future research should standardize and advance toward a dynamic hybrid monitoring paradigm, where subjective and objective measures are collected simultaneously and continuously, creating a personalized symptom fingerprint that neither method could achieve in isolation.

We acknowledge that high-precision laboratory instruments, such as force platforms and isokinetic dynamometers, remain the gold standard for biomechanical assessment [11]. However, their stationary nature limits them to capturing only a snapshot of the patient’s capacity in a controlled, artificial environment. This highlights the unique advantage of sensor-based monitoring: it prioritizes ecological validity to capture continuous, real-world data (eg, how a patient walks when tired, or how sleep quality affects morning stiffness) [10]. For the specific purpose of symptom monitoring, wearable sensors offer a vital complementary approach rather than a standalone solution. It is crucial to recognize that knee osteoarthritis symptoms are inherently multifactorial and driven by complex biopsychosocial mechanisms [35].

However, it is crucial to emphasize that the relationship between gross physical activity and multidimensional osteoarthritis symptoms is not strictly linear or inherently coherent. For instance, recent evidence demonstrates that in clinical knee osteoarthritis, the total volume of physical activity is often not associated with knee pain, functional decline, or health-related quality of life [36]. This lack of a direct correlation highlights a critical conceptual boundary: simple, isolated objective metrics, such as daily step counts, cannot serve as standalone proxies for the complex, biopsychosocial spectrum of osteoarthritis symptoms [26]. Instead, the true value of wearable technology lies in capturing context-dependent and qualitative markers [23,31], prioritizing ecological validity over the snapshot precision of stationary laboratory instruments like force platforms.

Consequently, wearable devices are not expected to perfectly correlate with or entirely replace subjective patient-reported outcomes. Instead, their expected validity lies in capturing the objective behavior and physiological correlates of these symptoms. By providing this continuous, objective dimension, wearables are expected to yield partial but clinically meaningful correlations with subjective experiences [26]. Future research should thus focus on using lab standards to validate these portable metrics, ensuring that this hybrid approach—integrating objective sensor data with subjective patient reports—maintains comprehensive clinical reliability [37].

Beyond biomechanical assessments, physiological sensors offer a vital systemic view of the disease burden. However, the specific pathophysiological pathways linking systemic physiological signals, such as HRV derived from ECG, to localized joint disease remain to be fully characterized [28]. Growing evidence suggests that knee osteoarthritis is associated with broader systemic responses, particularly regarding autonomic nervous system dysregulation and central sensitization [28,29]. Thus, physiological sensors may offer valuable insights into the systemic burden of pain, rather than the structural pathology of the joint itself. Therefore, wearable ECG and bioimpedance sensors are not intended to monitor the joint structure itself, but rather the physiological cost of pain—the systemic stress and inflammatory burden that patients experience [28,29]. By capturing these objective physiological markers, we can potentially identify patients who may benefit more from stress-management interventions than mechanical offloading [38].

However, it is important to note that monitoring itself does not constitute an intervention. The transition from passive data collection to active symptom management requires a combination of different technologies and strategies. Specifically, future research should explore integrating these continuous monitoring paradigms with just-in-time adaptive interventions in mobile health [39]. In this model, real-time objective sensor data and subjective reports act as tailored triggers to deliver personalized, timely behavioral support and stress-management interventions, thereby creating a true active feedback loop.

### Limitations

This scoping review provides a comprehensive synthesis of the current state of sensor-based monitoring technologies, categorized by their relevance to specific clinical symptoms. A key strength is the multidimensional approach, moving beyond pain to include fatigue and physiological stress. However, limitations exist. First, the heterogeneity of sensor modalities and study designs precluded a meta-analysis of sensor-symptom correlations. Second, most included studies were observational, limiting our ability to infer causality between sensor metrics and symptom changes. Third, we excluded non-English studies, which may have omitted relevant technological advancements from other regions. Finally, many free-living studies relied on short monitoring durations (eg,<7 d), which may not fully capture the long-term fluctuating nature of knee osteoarthritis symptoms.

### Conclusions

In conclusion, this scoping review synthesizes the current landscape of sensor-based monitoring for knee osteoarthritis by mapping technologies across a multidimensional symptom spectrum, including fatigue, sleep quality, and physiological stress. By constructing an evidence gap map, we identified a distinct saturation in biomechanical gait analysis alongside a critical gap in physiological monitoring. Crucially, the observed discrepancies between subjective and objective data underscore why neither assessment method is sufficient in isolation. The primary real-world implication of these findings is the necessity of a hybrid monitoring paradigm. For sensor technology to successfully translate into daily patient care, it must integrate objective ecological data with patient-reported outcomes to enable digital phenotyping—distinguishing patients driven by mechanical instability from those driven by systemic stress. Furthermore, while the current literature primarily consists of observational studies that successfully fulfill their aims of passive data collection, a vital future direction is the development of adaptive, event-based sampling, where sensor-detected anomalies trigger real-time subjective assessments. Ultimately, this hybrid approach will facilitate a shift from merely tracking disease progression toward supporting just-in-time adaptive interventions. By combining continuous monitoring with distinct interventional strategies, future systems can provide active, closed-loop feedback, thereby empowering patients to self-manage the complex and fluctuating nature of their condition.

## Supplementary material

10.2196/84262 Multimedia Appendix 1 Search terms.

10.2196/84262 Multimedia Appendix 2 Summary of characteristics for the 16 included studies evaluating sensor technologies for knee osteoarthritis. Data detail the specific sensor modalities used (eg, inertial measurement unit, electromyography, and electrocardiography), sensor placement, duration of monitoring in free-living settings, and the alignment between objective sensor metrics and subjective clinical symptoms (eg, pain, fatigue, and stiffness).

10.2196/84262 Checklist 1 PRISMA-ScR checklist.

## Abbreviations

ECG: electrocardiography
HRV: heart rate variability
IMU: inertial measurement unit
PPG: photoplethysmography
PRESS: Peer Review of Electronic Search Strategies
PRISMA-S: Preferred Reporting Items for Systematic Reviews and Meta-Analyses and search extension
PRISMA-ScR: Preferred Reporting Items for Systematic Reviews and Meta-Analyses extension for Scoping Reviews
PROM: patient-reported outcome measure
ROM: range of motion
sEMG: surface electromyography
