# Supplementary material for: Sensor-Based Monitoring of Knee Osteoarthritis Symptoms in Free-Living Settings: Scoping Review
Source: J Med Internet Res. 2026 Jul 2;28:e84262. doi: 10.2196/84262 (PMC13327683; doi:10.2196/84262)
Supplement: Multimedia Appendix 1 [file jmir-v28-e84262-s001.docx]

**Search Term**

PubMed

("Osteoarthritis, Knee"[Mesh] OR "Knee osteoarthritis"[Title/Abstract] OR "Knee OA"[Title/Abstract] OR Gonarthrosis[Title/Abstract]) AND ("Pain"[Mesh] OR "Pain"[Title/Abstract] OR Stiffness[Title/Abstract] OR Tenderness[Title/Abstract] OR Swelling[Title/Abstract] OR Symptom*[Title/Abstract] OR "Range of motion"[Title/Abstract] OR ROM[Title/Abstract] OR Function*[Title/Abstract]) AND ("Monitoring, Ambulatory"[Mesh] OR Monitor*[Title/Abstract] OR Track*[Title/Abstract] OR Measur*[Title/Abstract] OR Daily[Title/Abstract] OR Continuous[Title/Abstract] OR Ambulatory[Title/Abstract] OR "Free-living"[Title/Abstract] OR Assess*[Title/Abstract] OR Validat*[Title/Abstract] OR Evaluat*[Title/Abstract]) AND ("Wearable Electronic Devices"[Mesh] OR "Accelerometry"[Mesh] OR "Gait Analysis"[Mesh] OR "Biomechanical Phenomena"[Mesh] OR "Electrocardiography"[Mesh] OR "Mobile Applications"[Mesh] OR "Smartphone"[Mesh] OR Wearable*[Title/Abstract] OR Sensor*[Title/Abstract] OR Acceleromet*[Title/Abstract] OR IMU[Title/Abstract] OR "Inertial measurement unit"[Title/Abstract] OR Pedometer[Title/Abstract] OR "Gait analysis"[Title/Abstract] OR Biomechanic*[Title/Abstract] OR Kinematic*[Title/Abstract] OR PPG[Title/Abstract] OR Photoplethysmogra*[Title/Abstract] OR ECG[Title/Abstract] OR EMG[Title/Abstract] OR Electromyogra*[Title/Abstract] OR "Heart rate"[Title/Abstract] OR HRV[Title/Abstract] OR "Physiological signal*"[Title/Abstract] OR App[Title/Abstract] OR Apps[Title/Abstract] OR Smartphone*[Title/Abstract] OR mHealth[Title/Abstract] OR Goniometer[Title/Abstract]))

Web of Science

TS=("Knee osteoarthritis" OR "Knee OA" OR Gonarthrosis) AND TS = (Pain OR Stiffness OR Tenderness OR Swelling OR Symptom* OR "Range of motion" OR ROM OR Function*) AND TS=(Monitor* OR Track* OR Measur* OR Daily OR Continuous OR Ambulatory OR "Free-living" OR Assess* OR Validat* OR Evaluat*) AND TS=(Wearable* OR Sensor* OR Acceleromet* OR "Gait analysis" OR Biomechanic* OR Kinematic* OR IMU OR "Inertial measurement unit" OR Pedometer* OR PPG OR Photoplethysmogra* OR ECG OR EKG OR EMG OR Electromyogra* OR "Heart rate" OR HRV OR "Physiological signal*" OR App OR Apps OR Smartphone* OR mHealth OR Goniometer)

Embase

('knee osteoarthritis'/exp OR 'knee osteoarthritis':ti,ab,kw OR 'knee oa':ti,ab,kw OR 'gonarthrosis':ti,ab,kw) AND ('pain'/exp OR 'pain':ti,ab,kw OR 'stiffness':ti,ab,kw OR 'tenderness':ti,ab,kw OR 'swelling':ti,ab,kw OR 'symptom*':ti,ab,kw OR 'range of motion':ti,ab,kw OR 'rom':ti,ab,kw OR 'function*':ti,ab,kw) AND ('ambulatory monitoring'/exp OR 'monitor*':ti,ab,kw OR 'track*':ti,ab,kw OR 'measur*':ti,ab,kw OR 'daily':ti,ab,kw OR 'continuous':ti,ab,kw OR 'ambulatory':ti,ab,kw OR 'free-living':ti,ab,kw OR 'assess*':ti,ab,kw OR 'validat*':ti,ab,kw) AND ('wearable computer'/exp OR 'accelerometry'/exp OR 'gait analysis'/exp OR 'biomechanics'/exp OR 'electrocardiography'/exp OR 'electromyography'/exp OR 'mobile application'/exp OR 'smartphone'/exp OR 'wearable*':ti,ab,kw OR 'sensor*':ti,ab,kw OR 'acceleromet*':ti,ab,kw OR 'imu':ti,ab,kw OR 'inertial measurement unit':ti,ab,kw OR 'pedometer':ti,ab,kw OR 'gait analysis':ti,ab,kw OR 'biomechanic*':ti,ab,kw OR 'kinematic*':ti,ab,kw OR 'ppg':ti,ab,kw OR 'photoplethysmogra*':ti,ab,kw OR 'ecg':ti,ab,kw OR 'emg':ti,ab,kw OR 'electromyogra*':ti,ab,kw OR 'heart rate':ti,ab,kw OR 'hrv':ti,ab,kw OR 'app':ti,ab,kw OR 'apps':ti,ab,kw OR 'smartphone*':ti,ab,kw OR 'goniometer':ti,ab,kw)

IEEE

("All Metadata": "Knee osteoarthritis" OR "All Metadata": "Knee OA") AND ("All Metadata": Pain OR "All Metadata": Stiffness OR "All Metadata": Symptom* OR "All Metadata": ROM) AND ("All Metadata": Monitor* OR "All Metadata": Measur* OR "All Metadata": Daily OR "All Metadata": Assess*) AND ("All Metadata": Wearable* OR "All Metadata": Sensor* OR "All Metadata": Accelerometer OR "All Metadata": IMU OR "All Metadata": "Gait analysis" OR "All Metadata": PPG OR "All Metadata": EMG OR "All Metadata": App OR "All Metadata": Smartphone OR "All Metadata": Goniometer)
