# Supplementary material for: Sensor-Based Monitoring of Knee Osteoarthritis Symptoms in Free-Living Settings: Scoping Review
Source: J Med Internet Res. 2026 Jul 2;28:e84262. doi: 10.2196/84262 (PMC13327683; doi:10.2196/84262)
Supplement: Multimedia Appendix 2 [file jmir-v28-e84262-s002.docx]

## Appendix

### Search term

#### PubMed

("Osteoarthritis, Knee"[Mesh] OR "Knee osteoarthritis"[Title/Abstract] OR "Knee OA"[Title/Abstract] OR Gonarthrosis[Title/Abstract])

AND

("Pain"[Mesh] OR "Pain"[Title/Abstract] OR Stiffness[Title/Abstract] OR Tenderness[Title/Abstract] OR Swelling[Title/Abstract] OR Symptom*[Title/Abstract] OR "Range of motion"[Title/Abstract] OR ROM[Title/Abstract] OR Function*[Title/Abstract])

AND

("Monitoring, Ambulatory"[Mesh] OR Monitor*[Title/Abstract] OR Track*[Title/Abstract] OR Measur*[Title/Abstract] OR Daily[Title/Abstract] OR Continuous[Title/Abstract] OR Ambulatory[Title/Abstract] OR "Free-living"[Title/Abstract] OR Assess*[Title/Abstract] OR Validat*[Title/Abstract] OR Evaluat*[Title/Abstract])

AND

("Wearable Electronic Devices"[Mesh] OR "Accelerometry"[Mesh] OR "Gait Analysis"[Mesh] OR "Biomechanical Phenomena"[Mesh] OR "Electrocardiography"[Mesh] OR "Mobile Applications"[Mesh] OR "Smartphone"[Mesh] OR Wearable*[Title/Abstract] OR Sensor*[Title/Abstract] OR Acceleromet*[Title/Abstract] OR IMU[Title/Abstract] OR "Inertial measurement unit"[Title/Abstract] OR Pedometer[Title/Abstract] OR "Gait analysis"[Title/Abstract] OR Biomechanic*[Title/Abstract] OR Kinematic*[Title/Abstract] OR PPG[Title/Abstract] OR Photoplethysmogra*[Title/Abstract] OR ECG[Title/Abstract] OR EMG[Title/Abstract] OR Electromyogra*[Title/Abstract] OR "Heart rate"[Title/Abstract] OR HRV[Title/Abstract] OR "Physiological signal*"[Title/Abstract] OR App[Title/Abstract] OR Apps[Title/Abstract] OR Smartphone*[Title/Abstract] OR mHealth[Title/Abstract] OR Goniometer[Title/Abstract]))

#### Web of Science

TS=("Knee osteoarthritis" OR "Knee OA" OR Gonarthrosis)

AND

TS = (Pain OR Stiffness OR Tenderness OR Swelling OR Symptom* OR "Range of motion" OR ROM OR Function*)

AND

TS=(Monitor* OR Track* OR Measur* OR Daily OR Continuous OR Ambulatory OR "Free-living" OR Assess* OR Validat* OR Evaluat*)

AND

TS=(Wearable* OR Sensor* OR Acceleromet* OR "Gait analysis" OR Biomechanic* OR Kinematic* OR IMU OR "Inertial measurement unit" OR Pedometer* OR PPG OR Photoplethysmogra* OR ECG OR EKG OR EMG OR Electromyogra* OR "Heart rate" OR HRV OR "Physiological signal*" OR App OR Apps OR Smartphone* OR mHealth OR Goniometer)

#### Embase

('knee osteoarthritis'/exp OR 'knee osteoarthritis':ti,ab,kw OR 'knee oa':ti,ab,kw OR 'gonarthrosis':ti,ab,kw)

AND

('pain'/exp OR 'pain':ti,ab,kw OR 'stiffness':ti,ab,kw OR 'tenderness':ti,ab,kw OR 'swelling':ti,ab,kw OR 'symptom*':ti,ab,kw OR 'range of motion':ti,ab,kw OR 'rom':ti,ab,kw OR 'function*':ti,ab,kw)

AND

('ambulatory monitoring'/exp OR 'monitor*':ti,ab,kw OR 'track*':ti,ab,kw OR 'measur*':ti,ab,kw OR 'daily':ti,ab,kw OR 'continuous':ti,ab,kw OR 'ambulatory':ti,ab,kw OR 'free-living':ti,ab,kw OR 'assess*':ti,ab,kw OR 'validat*':ti,ab,kw)

AND

('wearable computer'/exp OR 'accelerometry'/exp OR 'gait analysis'/exp OR 'biomechanics'/exp OR 'electrocardiography'/exp OR 'electromyography'/exp OR 'mobile application'/exp OR 'smartphone'/exp OR

'wearable*':ti,ab,kw OR 'sensor*':ti,ab,kw OR 'acceleromet*':ti,ab,kw OR 'imu':ti,ab,kw OR 'inertial measurement unit':ti,ab,kw OR 'pedometer':ti,ab,kw OR 'gait analysis':ti,ab,kw OR 'biomechanic*':ti,ab,kw OR 'kinematic*':ti,ab,kw OR 'ppg':ti,ab,kw OR 'photoplethysmogra*':ti,ab,kw OR 'ecg':ti,ab,kw OR 'emg':ti,ab,kw OR 'electromyogra*':ti,ab,kw OR 'heart rate':ti,ab,kw OR 'hrv':ti,ab,kw OR 'app':ti,ab,kw OR 'apps':ti,ab,kw OR 'smartphone*':ti,ab,kw OR 'goniometer':ti,ab,kw)

#### IEEE

("All Metadata": "Knee osteoarthritis" OR "All Metadata": "Knee OA")

AND ("All Metadata": Pain OR "All Metadata": Stiffness OR "All Metadata": Symptom* OR "All Metadata": ROM)

AND ("All Metadata": Monitor* OR "All Metadata": Measur* OR "All Metadata": Daily OR "All Metadata": Assess*)

AND ("All Metadata": Wearable* OR "All Metadata": Sensor* OR "All Metadata": Accelerometer OR "All Metadata": IMU OR "All Metadata": "Gait analysis" OR "All Metadata": PPG OR "All Metadata": EMG OR "All Metadata": App OR "All Metadata": Smartphone OR "All Metadata": Goniometer)

| Category | Study Info & Design | | | | Population | | | | Technology & Protocol | | | | Symptom Assessment | | | Objective Markers & Key Findings | |
| --- | --- | --- | --- | --- | --- | --- | --- | --- | --- | --- | --- | --- | --- | --- | --- | --- | --- |
|  | Year | Country | Study Design | Environment | Sample size | Age | BMI | Sex(Female) | Sensor | Placement | Sampling rate(Hz) | Duration | Tool | Symptom | Frequency | Marker | Main finding |
| Biomechanical and Functional Changes [22] | 2022 | Japan | Cross-sectional Study | Lab | 20 | 60.0 ± 5.79 | 23.9 ± 3.26 | 15 | RGB-D | knee joint | 20 | One-time capture | VAS | pain | One-time | Knee joint trajectory | Early KOA patients showed RKJT was 10 mm larger than the control group (p=0.038), correlating with pain severity. |
| Biomechanical and Functional Changes [23] | 2021 | Japan | Cross-sectional Study | Lab | 21 | 24.9 ± 3.4 | 72.1 ± 7.10 | 17 | IMU | full-body | 60 | 5s | VAS | pain | One-time | Knee flexion excursion, muscle strength | As walking speed increased, stride length and knee flexion excursions significantly increased. Knee flexion score correlated significantly with pain score and muscle strength. |
| Biomechanical and Functional Changes [24] | 2022 | USA | Cross-sectional Study | Lab | 25 | 64±07 | 29±04 | 13 | IMU | lumbar | 128 | 2 min | KOOS | pain | One-time | Gait parameters | Gait velocity, cadence, step count, and stride length were the most important features for classifying KOA vs. pain levels. |
| Biomechanical and Functional Changes [25] | 2024 | USA | Cross-sectional Study | Lab | 28 | 68.7 ± 2.4 | 31.5 ± 1.6 | 18 | Cleveland Clinic configuration marker | full-body | 200 | One-time capture | WOMAC | pain | One-time | vGRF | Higher peak tibial acceleration is linked to worse knee pain (r=0.39; p=0.01) and higher vertical load rates. |
| Biomechanical and Functional Changes [26] | 2024 | USA | Cross-sectional Study | Lab | 42 | 60.4 ± 12.6 | 30.0 ± 6.8 | 28 | IMU | tibia | 1000 | 3 min | VAS | pain | One-time | peak tibial acceleration | An association exists between pain, obesity, and increased ankle-joint loading during the weight-acceptance phase of stair descent (p<0.001). |
| Biomechanical and Functional Changes [33] | 2020 | Belgium | Cross-sectional Study | Lab | 19 | 65.1 ± 5.2 | 26.0 ± 2.2 | 7 | IMU | lower limb joints | 60 | One-time capture | KOOS | pain, ADL | One-time | Joint Angles | IMU system successfully discriminated PwKOA from controls in all tasks except sit-to-stand. PwKOA showed reduced knee flexion ROM across walking, lunges, squats, and stairs (p=0.001). |
| Neuromuscular Control and Postural Stability [27] | 2025 | Greece | Cohort Study | Daily life | 21 | 51.0 ± 8 | NA | 13 | sEMG | quadriceps | 2000 | 8 weeks | WOMAC | pain | Daily | Muscle Performance | Significant Group×Time interaction for RMS (p<0.001). Increase in sEMG RMS (activation) coincided with a significant decrease in WOMAC pain scores. |
| Neuromuscular Control and Postural Stability [28] | 2021 | USA | Cohort Study | Lab | 1666 | 67.2 ± 7.6 | 32 ± 13 | 985 | sEMG | quadriceps and hamstrings | 1000 | One-time capture | WOMAC | pain | One-time | Muscle Co-activation | Significant inverse associations between hamstring coactivation and quadriceps strength. Lower quadriceps strength predicts incident KOA and pain. |
| Neuromuscular Control and Postural Stability [37] | 2025 | Malaysia | Cross-sectional Study | Lab | 64 | 69 ± 4 | 25.92 ± 3.25 | 19 | sEMG | lower limb joints | 9M | One-time capture | ultrasonic system | stiffness | One-time | Muscle Co-activation Index | Quadriceps stiffness was significantly greater in the KOA group and correlated with functional deficits. |
| Physical Activity Patterns [29] | 2022 | China | Cohort Study | Daily life | 65 | 61.3 ± 5.99 | 28.7 ± 28.66 | 30 | IMU | wrist | 60 | 7 days | WOMAC | pain | Daily | Steps | Weak but significant correlation between change in mean steps per day and global improvement / WOMAC function (p=0.08). |
| Physical Activity Patterns [30] | 2024 | UK | Proof of Concept | Daily life | 38 | 58 ± 9 | NA | 33 | IMU | Wrist | NA | 12 weeks | MSK-HQ | pain | Daily | Activity level | Significant improvements across all symptom domains (p<0.001). Largest effect sizes observed for fatigue (d=1.30) and day pain (d=1.03) following intervention. |
| Physiological Markers [31] | 2024 | Germany | Cross-sectional Study | Lab | 148 | 66 ± 27 | 30.6 ± 5.8 | 68 | ECG | chest | 60 | 5 min | PSQ-20 | pain | One-time | Heart rate variability | Weak negative correlation between HRV and pain. WOMAC pain significantly correlated with Cortisol (positive) and DHEA-S (negative). |
| Physiological Markers [32] | 2023 | USA | Cohort Study | Daily life | 10 | 73.5±8.26 | NA | 9 | Bioimpedance sensing | knee joint | 64 | 7 days | ESM | pain | Daily | Bioimpedance | Results suggest bioimpedance metrics can be used as a predictor for active pain experiences in knee OA. |
| Physiological Markers [34] | 2022 | USA | Cross-sectional Study | Lab | 30 | 58.3 ± 9.3 | 33.5 ± 5.8 | 20 | Portable gas exchange system | face | 10 | 10 min | KOOS | pain, fatigue | One-time | VO_2 peak, Heart Rate, Energy Cost | Higher energy cost for walking is linked to reduced physical activity. Higher fatigue and fatigability mediated the associations between walking energetics and activity. |
| Physiological Markers [36] | 2020 | Brazil | Cross-sectional Study | Lab | 11 | 63.1±9.5 | 28.7±4.0 | NA | Infrared sensor | thigh, leg | 30 | One-time capture | WOMAC, VAS | physical function, pain | One-time | Temperature | Affected knees had higher temperature, though not directly associated with pressure pain thresholds in this small sample. |
| Sleep and Circadian Interactions [35] | 2019 | USA | Cohort Study | Daily life | 160 | 71 ± 4 | 29.6 ± 3.4 | 99 | IMU | wrist | 60 | 5 days | WOMAC, BFI | pain, fatigue, sleep | Daily | Activity level, Sleep Duration | Contrast between subjective and objective data. Better subjective sleep predicted lower pain (p<0.001), but objective metrics showed no meaningful association with daily pain fluctuations. |

Table 1. Summary of characteristics for the 16 included studies evaluating sensor technologies for knee osteoarthritis. Data details the specific sensor modalities used (e.g., IMU, EMG, ECG), sensor placement, duration of monitoring in free-living settings, and the alignment between objective sensor metrics and subjective clinical symptoms (e.g., pain, fatigue, stiffness).

Abbreviations: ADL: Activities of Daily Living; BMI: Body Mass Index; ECG: Electrocardiography; ESM: Experience Sampling Method; HRV: Heart Rate Variability; IMU: Inertial Measurement Unit; KOA: Knee Osteoarthritis; KOOS: Knee Injury and Osteoarthritis Outcome Score; MSK-HQ: Musculoskeletal Health Questionnaire; PSQ-20: Perceived Stress Questionnaire; PTA: Peak Tibial Acceleration; RGB-D: Red Green Blue-Depth; RMS: Root Mean Square; ROM: Range of Motion; sEMG: Surface Electromyography; TMCf: Time of Muscle Co-activation function; VAS: Visual Analog Scale; vGRF: Vertical Ground Reaction Force; WOMAC: Western Ontario and McMaster Universities Osteoarthritis Index.
