# Supplementary material for: Sensor-Based Monitoring of Knee Osteoarthritis Symptoms in Free-Living Settings: Scoping Review
Source: J Med Internet Res. 2026 Jul 2;28:e84262. doi: 10.2196/84262 (PMC13327683; doi:10.2196/84262)
Supplement: Checklist 1 [file jmir-v28-e84262-s003.docx]

| **No.** | **Section** | **Item Description** | **Reported on Page** |
| --- | --- | --- | --- |
| 1 | Title | Identify the report as a scoping review. | Title page |
| 2 | Abstract | Provide a structured summary that includes background, objectives, eligibility criteria, data sources, charting methods, and main results. | Abstract |
| 3 | Introduction: Rationale | Describe the rationale for the review in the context of what is already known. | p.2 |
| 4 | Introduction: Objectives | Provide an explicit statement of the questions and objectives being addressed. | p.2-3 |
| 5 | Methods: Eligibility criteria | Specify characteristics of sources of evidence (e.g., years considered, language, publication status) used as inclusion/exclusion criteria. | p.4-5 |
| 6 | Methods: Information sources | Describe all information sources (e.g., databases with dates of coverage). | p.3 |
| 7 | Methods: Search | Present the full electronic search strategy for at least one database. | p.3-4, Appendix |
| 8 | Methods: Selection of sources | State the process for selecting sources of evidence (e.g., screening, eligibility, included). | p.3-4 |
| 9 | Methods: Data charting process | Describe the methods of charting data from the included sources. | p.6 |
| 10 | Methods: Data items | List and define all variables for which data were sought. | p.6 |
| 11 | Methods: Critical appraisal of sources (optional) | If done, provide a rationale and describe the methods used for assessing quality. | Not applicable |
| 12 | Methods: Synthesis of results | Describe the methods used to handle and summarize the charted data. | p.6 |
| 13 | Results: Selection of sources | Provide the number of sources screened, assessed for eligibility, and included, with reasons for exclusions. | Figure 1 |
| 14 | Results: Characteristics of sources | Present characteristics of each source of evidence. | Table I |
| 15 | Results: Charting results | Present relevant data charted from the included studies. | Table I |
| 16 | Results: Synthesis of results | Summarize the charting results as they relate to the review questions. | p.7–12 |
| 17 | Discussion: Summary of evidence | Summarize main findings, their implications, and relevance to key groups. | p.12–14 |
| 18 | Discussion: Limitations | Discuss the limitations of the scoping review process. | p.14 |
| 19 | Conclusions | Provide a general interpretation of the results with respect to the review questions. | p.13-14 |
| 20 | Funding | Describe sources of funding and the role of funders. | Acknowledgements |
